# Do Couple-Based Interventions Show Larger Effects in Promoting HIV Preventive Behaviors than Individualized Interventions in Couples? A Systematic Review and Meta-analysis of 11 Randomized Controlled Trials

**DOI:** 10.1007/s10461-022-03768-5

**Published:** 2022-07-15

**Authors:** Rong Fu, Jianhua Hou, Yuzhou Gu, Nancy Xiaonan Yu

**Affiliations:** 1 Department of Social and Behavioural Sciences, City University of Hong Kong, Hong Kong, People’s Republic of China; 2 Guangzhou Center for Disease Control and Prevention, Guangzhou, People’s Republic of China

**Keywords:** Couple-based intervention, HIV, Meta-analysis, Prevention, Randomized controlled trials (RCTs), Intervención basada en pareja, VIH, metanálisis, prevención, ensayos controlados aleatorios (ECA)

## Abstract

This systematic review and meta-analysis aims to compare the effects of couple-based prevention interventions against individual-level interventions on HIV prevention in randomized controlled trials (RCTs), identify potential moderators, and assess study quality. Eleven RCTs were included, comprising 3933 couples in the intervention group and 7125 individuals in the individual control group, predominantly in heterosexual couples from the USA and Africa. Couple-based interventions had a more significant effect in promoting condom use and HIV testing. Education levels of high school or above, residence in low- and middle-income countries, and intervention design incorporating HIV counseling and testing were associated with higher odds of condom use. The quality assessment analysis identified methodological and theoretical heterogeneity factors. Evidence of couple-based HIV prevention RCTs among men who have sex with men, injecting drug users, sex workers, and transgender women warrant further investigation. Recommendations are made to improve the quality and replicability of future intervention studies.

**Supplementary Information:**

The online version contains supplementary material available at 10.1007/s10461-022-03768-5.

## Resumen

Esta revista sistemática y metanálisis tiene por objeto comparar los efectos de las intervenciones de prevención basadas en pareja con las a nivel individual en la prevención del VIH en ensayos controlados aleatorios (ECA), identificar posibles moderadores y evaluar la calidad de los estudios. Se incluyeron once ECA, que comprendían 3.933 parejas en el grupo de intervención y 7.125 personas en el grupo de control individual, la mayoría de las cuales eran heterosexuales de EE.UU. y de África. Las intervenciones basadas en pareja son más eficaces para promover el uso de preservativos y pruebas del VIH. Los niveles de educación secundaria o superior, los países de ingresos bajos y medianos, y los diseños de intervención que incluyen pruebas y asesoramiento sobre el VIH se relacionan con más uso de preservativos. El análisis de evaluación de la calidad ha identificado factores de heterogeneidad metodológica y teórica. Las pruebas de ECA basadas en pareja para la prevención del VIH en hombres que tienen sexo con hombres, consumidores de drogas inyectables, trabajadoras sexuales y mujeres transgénero necesitan más investigación. Se hacen recomendaciones para mejorar la calidad y replicabilidad de futuros estudios de intervención.

## Introduction

Sexual transmission of HIV between two sexually intimate partners remains the primary cause of the HIV epidemic in many parts of the world [1]. In this specific micro-social situation, dissonance between partners emerges in the liaison of interaction and interdependence [2]. This creates a variegated picture of the sexual risks driving HIV transmission among couples [3]. Partner bonds may be resilient through shared resources and coping efforts conducive to one or both individuals’ improved health outcomes [4]. However, the intimate bond between couples can complicate efforts to sustain HIV transmission prevention methods. A good illustration is HIV prevention between serodiscordant partners in which one partner has positive HIV status; even though HIV transmission is a persistent health threat to serodiscordant couples, they may not use condoms consistently [5] because condom use delegitimizes the trust and intimacy of a romantic relationship [6]. In such situations where individual resources are overwhelmed, partners can prevent HIV transmission while promoting trust and intimacy by calling on shared resources and efforts, including sexual negotiation [7–9], virus load suppression for the HIV positive partner (i.e., the outlook of “undetectable equals untransmittable” status; i.e., U = U) [8, 10, 11], as well as HIV counseling and testing [12], and access to pre-exposure prophylaxis (PrEP) [13] for the HIV-negative partner.

The past two decades have witnessed growing advancement in couple-based HIV prevention programs. Recent empirical evidence supports the credibility of couple-based interventions, particularly when compared to individual-level interventions with large base sizes. A recent meta-analysis suggested that couple-based interventions are more effective than individual-level interventions in promoting HIV-protective behaviors to prevent HIV transmission and infection [14]. Dyadic perspectives may increase couples’ sense of shared responsibility for HIV and sexually transmitted infections (STI) prevention, motivating them to work together to stay healthy and reinforcing intimate partner bonding by treating two partners as an intervention unit. This approach is advantageous in offering preventive interventions for HIV and other STI because it delivers practical skills in communication and negotiation for safer sex.

Parallel to this, the development of HIV-prevention randomized controlled trials (RCTs) in key populations (i.e., injecting drug users, sex workers, gay men and other men who have sex with men [MSM], and transgender populations) and their sexual partners has become an overarching public health issue. One important observation is that in recent years, more than 60% of new adult HIV cases worldwide occurred in key populations [1]. However, because they face persistent health inequities, these populations have no, or very limited, access to HIV-related health resources [1]. It therefore becomes important that intervention programs not only help to mobilize resources or encourage the use of shared resources amongst key populations, but also ascertain the socioenvironmental factors that may inhibit these populations from implementing them. Another important point is that, with RCTs as the gold standard of evidence for intervention studies in the twenty-first century [15], using RCTs to evaluate the intervention effects of improving the health and well-being of key populations in the face of widespread global health inequities is now one of many pressing public health concerns [16].

To date, five systematic reviews and meta-analyses of HIV couple-based intervention and prevention studies have been published, which primarily provide qualitative and quantitative assessments of key intervention elements and study outcomes [14, 16–18]. However, there are two significant issues of heterogeneity limiting these studies: issues in the methodological design of empirical intervention studies and quality assessments of the meta-studies.

First, the scarcity of couple-based HIV prevention interventions has led to notable heterogeneity in study designs and outcomes in these five systematic reviews and meta-analyses. Although one of the five systematic reviews and meta-analyses compares couple-based and individual-level interventions [14], it achieves this only by mixing controlled trials and prospective cohort designs, thereby obfuscating the underlying intervention effects due to considerable selection bias in study design. However, RCTs may counter the heterogeneity seen in previous systematic reviews and meta-analyses more effectively by controlling self-selection and self-reporting bias in study design. Therefore, a meta-analysis of RCTs involving couple-based HIV prevention interventions will help clarify the effects of couple-based interventions over individual-level interventions.

Furthermore, methodological heterogeneity in HIV prevention intervention research may also be perceived on a theoretical level. Couple-based research frequently relies on individual-level theories as design and interpretation frameworks [16], potentially diminishing significant moderators and health outcomes of dyadic interactions and decision-making. Therefore, the development of dyad-level theories may more accurately explain inter- and intrapersonal interaction as well as the influence of health behaviors in couples [3, 19] by considering both partners’ emotions, cognition, and behaviors. These dyadic models may provide a more robust theoretical underpinning for research and intervention in addressing the needs of an intimate-partner relationship [20].

Second, there is distinct heterogeneity in the quality assessments used to substantiate each study’s internal validity among these five meta-studies. Only Jiwatram-Negrón and El-Bassel’s study [16] used an adapted assessment tool to provide quality assessment. A scarcity of accredited quality assessment guidelines may contribute to the lack of quality assessments conducted in the additional four studies. The Template for Intervention Description and Replication (TIDieR) guide published in 2014 clarifies intervention reporting to ensure the quality and replicability of intervention studies [21]. However, the five meta-studies above were conducted before the release of the TIDieR guide, potentially contributing to their lack of rigorous and uniform quality assessment. Thus, the present study facilitates the development of future intervention studies of couple-based HIV prevention by implementing a more comprehensive quality assessment framework through the integration of methodological and reporting quality assessments.

Considering the heterogeneity within the design, outcome, and quality assessment variables of previous meta-studies, this systematic review and meta-analysis aims to (a) estimate the relative magnitude of couple-based interventions over individual-level interventions’ effects on HIV preventive behaviors of sexual-risk reduction through direct comparison of RCTs; (b) identify potential moderators, particularly intervention- and relationship-specific factors of the intervention effect; and (c) assess the methodological and reporting quality of the intervention. This paper expands on existing systematic reviews and meta-analyses, integrating more recent literature with more robust methodological support and making suggestions for quality assessment.

## Methods

### Protocol Registration

This systematic review and meta-analysis was registered in the PROSPERO database (CRD42020222819, https://www.crd.york.ac.uk/PROSPERO/). We conducted and reported our systematic review and meta-analysis according to Preferred Reporting Items for Systematic Reviews and Meta-Analysis (PRISMA, [22]; Table A in Online Attachment).

### Searching Strategy

The first two authors (RF & JH) independently searched five electronic databases (Web of Science, PubMed/Medline, PsycInfo, CINAHL, and clinicaltrials.gov). The keywords combined participant (Couple OR dyad OR partner OR married), intervention-related terms (training OR intervention OR prevention), and disease-related terms (HIV OR AIDS) (See detailed search strings in Table B in Online Attachment). We also searched the reference lists of the published systematic review and original articles. All searches were limited to English peer-reviewed journal articles.

### Study Eligibility

Studies were eligible if they met the following criteria: (1) used RCT study design; (2) evaluated a couple-based preventive intervention compared to an individual-level control, including biobehavioral components (i.e., skills-building, HIV voluntary testing and counseling and antiretroviral therapy [ART] adherence) as previously categorized by Jiwatram-Negrón and El-Bassel [16]; (3) conducted in same-sex or heterosexual couples; and (4) measured pre- and post-intervention changes in at least one HIV-preventive behavior. Studies were excluded if they were: (1) a theoretical article or research protocol or (2) not peer-reviewed (i.e., an unpublished thesis, dissertation, or book chapter).

**Fig. 1:**
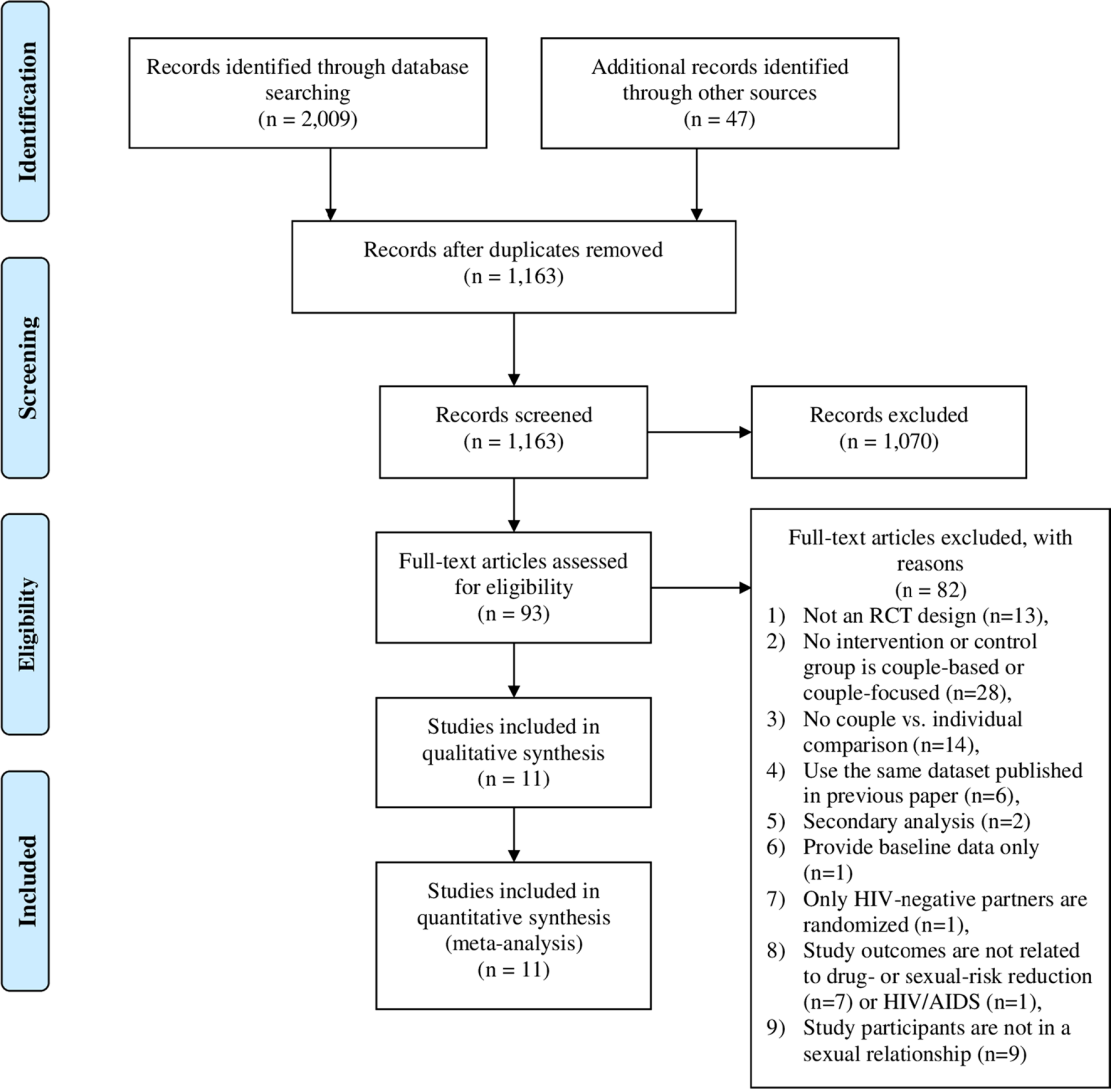
The flow chart of the included studies

Two authors (RF & JH) independently screened the results based on titles, abstracts, and full texts. RF & JH held three discussion meetings with the corresponding author (NXY) to resolve any discrepancies during the screening phase. The full texts were excluded if they (1) lacked RCT design, (2) lacked a couple-based or couple-focused intervention or control group, (3) lacked couple vs. individual comparison, (4) used the same dataset published in a previous paper, (5) used a secondary analysis, (6) provided only baseline data, (7) randomized only HIV-negative partners, (8) described study outcomes that were not related to sexual-risk reduction or HIV/AIDS, or (9) invited study participants who were not in a sexual relationship (Table C in Online Attachment). The selection process is shown in Fig. 1.

### Data Coding and Extraction

The outcomes of interest are HIV-preventive behaviors, including condom use (defined as consistent condom use or no condomless sex), HIV testing, STI reduction, and ART adherence. ART adherence can be measured by either a self-report scale or blood-based tests.

We (RF & JH) coded the adjusted odds ratios (aOR) with 95% confidential interval, means and standard deviations (SD) in each arm before and after interventions, and mean changes with variance in each arm or other data format used to calculate odds ratio.

We (RF & JH) also used a Shimo spreadsheet (https://shimo.im/desktop) to extract participant-related information (i.e., age, education attainment, sex distribution, type of couple, and relationship status), intervention-related data (i.e., sessions, frequency, total hours, intervention content, the format of control, and type of control), and implementation-related characteristic (i.e., fidelity and tailoring).

Specifically, we coded the type of couple into same-sex, heterosexual, and mixed. Relationship status is operationalized as the median or mean years of partnership or percentage of participants who regarded themselves as in a relationship.

### Statistical Analysis

First, a random-effects model was conducted using the odds ratio (OR) to estimate the comparative effect of the couple-based intervention on HIV preventive behavior. An OR higher than 1 indicated greater odds of condom use in the couple-based intervention than in the individual-level control. Forest plots illustrate the effect sizes for individual and pooled studies.

Second, we used Cochrane *Q* tests and *I*^2^ to assess the significance and proportion of the between-study heterogeneity [23]. Regarding publication bias, we utilized Egger’s intercept test to assess the asymmetry of the funnel plot [24].

Third, subgroup analysis and meta-regression were used to evaluate the source of heterogeneity if more than 10 comparisons were analyzed in any outcomes. In addition to a continuous variable of age, moderators of categorical variables included the education level (high school or above vs. below high school), intervention component (skills-building vs. HIV testing and counseling), type of couple (i.e., heterosexual, same-sex, or mixed), study quality (high vs. low; we compared studies with high bias against those with low bias to check the robustness of our findings [25]), total sessions (< 6 sessions vs. ≥ 6 sessions), tailoring (no or unclear vs. yes), fidelity (no or unclear vs. yes), HIV status (serodiscordant couple vs. negative or unknown), and country (high-income vs. low- and middle-income). Because the studies have relatively small sample sizes and/or are few, a *p* value of 0.1 was used, as suggested by the Cochrane handbook [26], to detect the significance of heterogeneity and subgroup analyses. Additionally, subgroup analysis was conducted after we found 10 or more comparisons of outcomes of interest [26].

All analyses were conducted in Comprehensive Meta-analysis Version 3 (CMA 3.0).

### Study Quality

Referring to previous meta-analyses concerning the effect of psychological interventions [25, 27], we used a modified Physiotherapy Evidence Database (PEDro) scale to evaluate the study quality, including participant eligibility, randomization, the comparable baseline in key outcomes, concealment, retention rate (> 85%) intention-to-treat (ITT), blindness to participants, between-group comparisons, and point estimates with variance [32]. Studies were regarded as high-quality if they scored 7 or higher (maximum score = 9). The first two authors independently checked the study quality point-to-point (Table D in Online Attachment). We also used a modified template for intervention description and replication (TIDieR) checklist to label whether the researchers have reported their intervention properly, which is crucial for study replication [21].

## Results

### Characteristics of Included Studies

Altogether, 11 eligible RCTs [28–38] were included in this meta-analysis (Table 1), comprising 3933 couples in the intervention group and 7125 individuals in the individual-level control, with heterosexual couples in the USA and Africa predominating. The mean participant age ranged from 18 to 45. Heterosexual couples were recruited exclusively in 10 out of 11 RCT studies, of which only one study covered injecting drug users and their sexual partners. Apart from this, only one of the 11 studies recruited male couples [38]. Three studies recruited serodiscordant couples [31, 35, 38] whereas others recruited seroconcordant HIV-negative or couples with unknown HIV status. The education level varied across studies, and the female proportion ranged from 0 to 72.8%. Six studies defined a couple by the length of the relationship (i.e., a minimum of being together for six months [28, 30–33, 35]). Only three of the included RCTs reported significant effects on HIV-preventive behaviors [29, 31, 36].

**Table 1:** Description of participant and relationship characteristics of 11 included studies

Study name	Participant characteristics				Relationship characteristics	
	No. of participants	Age	Education level	Female proportion	Type of couple	Relationship status
Becker et al. (2010) [28]	760 couples in voluntary counseling and testing for couples (CVCT) and 761 individuals in individual VCT (IVCT)	Mean = 24.8 years	Mean = 8 years	66.7%	Heterosexual couples	Legal marriage, traditional marriage, or living with the same partner for at least 2 years
Coates et al. (2000) [29]	589 couples in CVCT and 1563 individuals in IVCT	Mean = 29 years	< 6 years	50%	Heterosexual couples with unknown HIV status	N/A
El-Bassel et al. (2003) [30]	81 couples in couple intervention, 146 individuals in women-alone	> 25 years	< 12 years	72.8%	Heterosexual couples	Involvement with this partner for the past 6 months and intent to stay with him for at least 1 year
El-Bassel et al. (2010) [31]	260 couples in risk-reduction group and 550 individuals in health promotion group	Mean = 43.4 years	< 9 years	50%	HIV-1-serodiscordant heterosexual couples	> 6 months
El-Bassel et al. (2011) [32]	95 couples in risk reduction and 92 individuals in individual risk reduction	Mean = 36.5 years	Mean = 11.6 years	50%	Heterosexual couples	> 6 months
Jones et al. (2013) [33]	104 couples in experimental condition and 224 individuals in individual condition	Mean = 45 years	Mean = 11 years	50%	Heterosexual couple	> 6 months
McMahon et al. (2013) [34]	110 couples in CHTC (Couple-based HIV testing and counseling), 110 in WRF-HIV-CT (Women-only relationship-focused HIV testing and counseling)	Mean = 38.4 years	52.2% > high school	50%	Heterosexual couple	Mean = 7.62 years
Remien et al. (2005) [35]	106 couples in intervention and 109 individuals in individual control	Mean = 42 years	< high school	37%	HIV-serodiscordant Heterosexual and same-sex couple	> 6 months
Sharma et al. (2020) [36]	1692 couples in couples' UBL (Unite for a Better Life), 1707 individuals in women-only UBL, and 1691 individuals in men-only UBL,	Mean = 34.5 years	Most were none schooling	50%	Heterosexual couple	Mean = 10–15 years marriage
Speizer et al. (2018) [37]	98 couples in the CHC (Couples Health CoOp) and 84 individuals in the WHC (Women's Health CoOp) women-intervened-only comparison	Mean = 25.275 years	> High school	50%	Heterosexual couple	27.26% married or cohabiting
Sullivan et al. (2014) [38]	38 couples in CHCT and 88 individuals in IHCT	18–29 years	> high school	0	HIV-serodiscordant sex minority couple	Mean = 14 months

### Comparing Couple-Based Versus Individual-Based Intervention on HIV-Preventive Behaviors

#### Condom Use

**Fig. 2:**
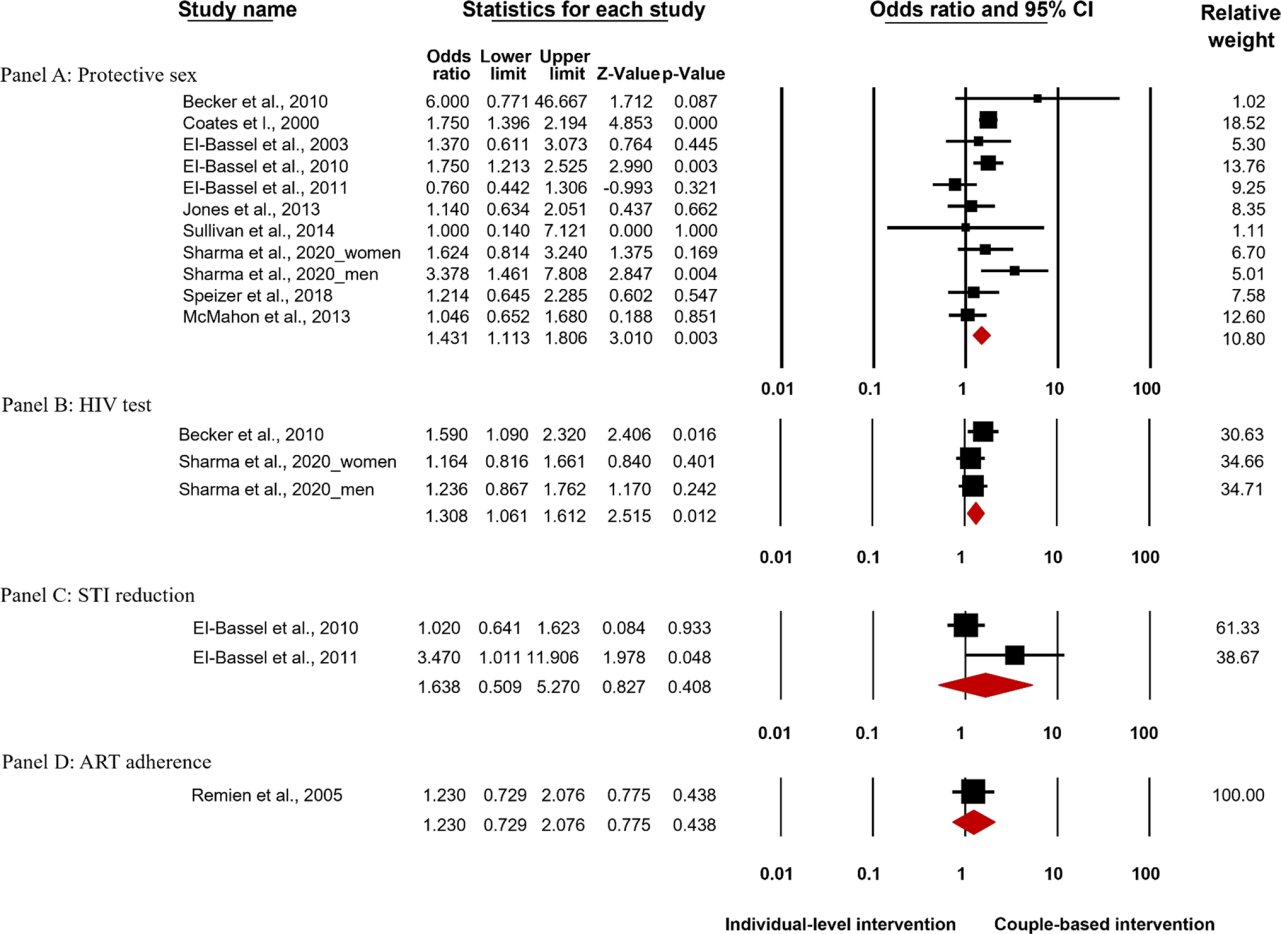
The forest plot for HIV preventive behaviors. Panel A: The pooled effect size for condom use. Panel B: The pooled effect sizefor HIV testing. Panel C: The pooled effect size for STI reduction. Panel D: The pooled effect size for ARTadherence

Eleven comparisons reported condom use during anal or vaginal sex acts. The composite effect size for the change of condom use between the two groups was 1.431 (95% CI 1.133–1.808, *p* = 0.003, random-effects model, Fig. 2, Panel A). No significant heterogeneity was detected across comparisons [*Q*(10) = 17.625, *p* = 0.062, *I*^2^ = 43.261%]. No significant asymmetry was detected from the funnel plot (Intercept = − 0.277, *p* = 0.742).

#### HIV Testing

Three comparisons reported HIV testing rates between the two groups. The composite effect size for the change in HIV testing between the two groups was 1.308 (95% CI 1.061–1.612, *p* = 0.012, Fig. 2, Panel B). No significant heterogeneity was detected across comparisons [*Q*(2) = 1.538, *p* = 0.464, *I*^2^ = 0]. No significant asymmetry was detected from the funnel plot (Intercept = 24.183, *p* = 0.120).

#### STI Reduction and ART Adherence

Two comparisons reported STI rates between the two groups. The composite effect size for the change of STI between the two groups was 1.638 (95% CI 0.509–5.270, *p* = 0.408; Fig. 2, Panel C). A significant heterogeneity was detected across comparisons [*Q*(1) = 3.318, *p* = 0.069, *I*^2^ = 69.86]. Only one study reported ART adherence between two groups with a non-significant effect size (*OR* 1.230, 95% CI 0.729–2.076, *p* = 0.438; Fig. 2, Panel D).

### Moderating Effects

Participants with an education level of high school or above showed a higher odds of condom use [*Q*(1) = 4.401, *p* = 0.036], compared to those had received less than a high school education. Compared to skills-building, interventions incorporating an HIV counseling and testing component were more effective in improving condom use [*Q*(1) = 3.275, *p* = 0.070]. Improvements in condom use were higher in low- and middle-income countries (LMICs) compared to studies conducted in high-income countries [*Q*(1) = 3.679, *p* = 0.054].

Other moderators—such as age, type of couple, HIV status, study quality, intervention sessions, tailoring, and fidelity—were not significantly associated with the intervention effect of condom use (Table 2). The number of trials was not sufficient to conduct a moderating analysis for the outcomes of HIV testing, STI reduction and ART adherence.

**Table 2:** Subgroup and meta-regression results for comparison of condom use between couple-based and individual-based interventions

Moderators	Number of comparisons	OR or coefficient	95% CI	*Q*	*p*
Age		− 0.010	− 0.040 to 0.020	0.570	0.450
Education level				4.401	**0.036**
Below high school	5	1.242	0.981 to 1.571		
High school or above	6	1.716	1.419 to 2.075		
Intervention component	11			3.275	**0.070**
Skills-building	8	1.339	1.100 to 1.631		
HIV testing and counseling	3	1.763	1.410 to 2.204		
Type of Couple	11			0.171	0.680
Heterosexual	10	1.514	1.306 to 1.750		
Same-sex or mixed	1	1.000	0.140 to 7.121		
Study quality	11			0.974	0.324
Low	4	1.245	0.825 to 1.879		
High	7	1.554	1.327 to 1.821		
Total sessions	11			1.027	0.311
< 6 sessions	5	1.617	1.327 to 1.972		
≥ 6 sessions	6	1.387	1.111 to 1.731		
Tailoring	11			0.249	0.681
No or unclear	5	1.637	1.154 to 2.323		
Yes	6	1.484	1.261 to 1.747		
Fidelity	11			2.189	0.139
No or unclear	4	1.692	1.371 to 2.088		
Yes	7	1.353	1.100 to 1.665		
Country				3.697	**0.054**
High-income	6	1.239	0.991 to 1.550		
Low- and middle-income	5	1.761	1.446 to 2.144		

Bold values are statistically significant (*p* < 0.1)

### Methodological and Reporting Quality

Online Table D summarizes the methodological quality of the included studies. All the included studies provided clear information on their eligibility criteria, randomization, similar baseline, and utilized appropriate statistical analysis for between-group comparisons [28–38]. However, six of the 11 studies lacked concealed allocation [28, 32–35, 37], seven studies did not blind the assessors [28–30, 32–34, 37], three studies did not analyze their data following ITT strategies [33, 37, 38], one study failed to obtain data from more than 85% of the participants initially allocated to both groups [38], and one study did not report the point estimate and its variance [28].

Table 3 summarizes the reporting quality of the included studies. Only one study reported the intervention as per the TIDieR checklist [36]. Seven out of 11 studies articulated their research aims using theoretical frameworks—social cognitive theory and combined ecological theory being the most frequently adopted [30–35, 37]. All studies except one did not report the provider(s) (e.g., trained counselor or facilitator) of the intervention [37]. Three studies were conducted in LMICs [28, 36, 37], seven in one high-income country (i.e., the USA [30–35, 38]), and one comprising data from three countries [29].

**Table 3:** Description of intervention characteristics in included studies, adopting the items from the templates for intervention description and replication (TIDieR) checklist

Study name	Brief name	Why	What (procedures)	Who provided	Where	When and how much	Tailoring	Strategies to improve or maintain intervention fidelity	Extent of intervention fidelity
Becker et al. (2010) [28]	CVCT vs. IVCT at antenatal clinics	Determine the acceptance and effectiveness of CVCT as compared to IVCT in preventing HIV transmission Guiding theory not specified	Antenatal visit with husband or wife only HIV counseling and testing Results of HIV testing (couple/wife only/separately) Antiretroviral treatment for all HIV-positive participants	Counselors trained with couple counseling strategies	3 antenatal clinics in in the Temeke district of Dar es Salaam, Tanzania	One-off CVCT/IVCT for 1 h	N/A	N/A	Completion of VCT: CVCT arm: 39% (294/760), IVCT arm: 71% (538/761)
Coates et al. (2000) [29]	CVCT vs. IVCT with health information control arms	Determine the efficacy of HIV-1 VCT in reducing unprotected sex among couples and individuals Guiding theory not specified	VCT arm: HIV-1 serum test Health-information arm: 15 min video and a discussion on HIV-1 transmission and condom use	Counselors and health-information officers trained with standard training manual	3 sites: Nairobi, Kenya Dar-Es-Salaam, Tanzania and Port-of-Spain, Trinidad	One-off CVCT/IVCT/health information intervention for 1 h	VCT was based on the US CDC's client-centered HIV-1 counseling model, including personalized risk assessment, development of a personalized risk-reduction plan for each client, and is ideal for promoting cultural specificity	N/A	Retention rate at second follow-up: CVCT arm: 76.1% (896/1178), IVCT arm: 70.7% (1105/1563)
El-Bassel et al. (2003) [30]	Project Connect	Aims: (1) Test the efficacy of a 6-session HIV/STD relationship-based intervention in sexual risk reduction among heterosexual couples as compared with a single session of HIV/STD education (2) Examine the efficacy of the intervention by comparing the couple-based group and woman only group Guiding theory: The AIDS Risk Reduction Model & the ecological perspective	(1) 1 orientation session and 5 relationship-based sessions (2) A single session of HIV/STD education	Sex-matched facilitators conducted orientation sessions and female facilitators conducted other sessions	Hospital-based outpatient clinics in Bronx, New York, USA	(1) Active conditions: 6 weekly 2-h intervention sessions for 6 weeks (2) Control condition: A single 1-h HIV/STD educational control session	N/A	Facilitators: Completed standardized training, used structured intervention protocols, had weekly meeting with clinical and task supervisors On-site supervisor: Routine monitoring and feedback Independent raters: Reviewed a random sample of 10% of the sessions for each facilitator for assessment of quality assurance (QA)	Intervention attendance rate (≥ 1 session) Couple sessions: 89% (72/83), Woman-alone sessions: 93% (68/73)
El-Bassel et al. (2010) [31]	Eban HIV/STD Prevention Intervention	Determine if a behavioral intervention can reduce sexual risk behaviors among African American HIV serodiscordant couples Guiding theory: Social Cognitive Theory	(1) Eban: 4 sessions with individual couples and 4 with groups of couples, focused on HIV/STD risk (2) Health promotion: structurally like Eban, but focused on behaviors linked to risk of heart disease, hypertension, stroke, and certain cancers	Male and female African American cofacilitator trained in HIV prevention	4 sites: Atlanta, Georgia; Los Angeles, California; New York, New York; and Philadelphia, Pennsylvania, USA	8 weekly 2-h intervention sessions over 8 weeks	The Eban is built on HIV prevention research with couples and high-risk African Americans, a culturally congruent couple-focused HIV/STD risk-reduction intervention	Facilitators: Used structured implementation protocols, completed fidelity assessment forms after each session, had weekly meeting with supervisors Supervisors: Reviewed audio-taped sessions and give feedback to facilitators An independent QA monitor: Rated the fidelity of a random sample of 10% of sessions from each intervention	Intervention attendance rate (8 sessions) RR group: 85.8% (223/260), Health promotion group: 76.0% (209/275)
El-Bassel et al. (2011) [32]	Project Connect 2	Determine the "intervention effect" and "modality effect" among the three conditions in decreasing sexual risk behaviors and incidence of STI Guiding theory: Social Cognitive Theory & A relationship-oriented ecological framework	(1) Active conditions: To validate the relationship's strengths of commitment, love, trust, and empower the dyad to enact protective behaviors (2) Couple wellness promotion: structurally like active conditions, but focused on maintaining a healthy diet, promoting physical fitness, promoting age-appropriate recommendations for screening for common diseases, and improving access to health care services	A single female or male facilitator matched to the gender of the index participant to enact preventive behavior	New York, New York, USA	7 weekly 2-h sessions over 7 weeks	Building on Project Connect, intervention components were modified to address dyadic drug risk reduction and drug-related unsafe sex	Recordings and session-specific QA checklists were reviewed to monitor fidelity of implementation and to provide feedback to facilitators	Intervention attendance rate (7 sessions) Couple risk reduction: 76% (145/190), Individual risk reduction: 66% (61/184), Couple wellness promotion: 72% (137/190)
Jones et al. (2013) [33]	The NOW2 study	Examine the impact of substance use, history of sexual trauma, and intimate partner violence on the sexual risk associated with participation in a risk reduction intervention Guiding theory: Theory of Reasoned Action & Theory of Planned Behavior	(1) Group intervention to increase couples' skills in sexual risk reduction, condom negotiation, and conflict resolution strategies (2) Individual intervention focused on HIV testing and counseling and behavioral changes, supplemented with HIV-related health education videos	Female and male facilitator trained by a clinical psychologist	Urban Miami-Dade County, South Florida, USA	4 weekly 2-h sessions over 4 weeks	N/A	Digital QA recording and session-specific QA checklists were reviewed by a clinical psychologist to monitor fidelity to condition and provide feedback to facilitators	Overall post-intervention retention rate: 87.5% (189/216)
McMahon et al. (2013) [34]	Harlem River Couples Project	Determine the "intervention effect" and "modality effect" among the three conditions in risk reduction outcomes Guiding theory: Social Cognitive Theory, Information-Motivation-Behavior Skills model, Stages-of-Change model, Theory of Gender and Power	(1) Active conditions: In the CB-HIV-CT group, intervention content was divided into pre and posttest sessions, including a short dyadic risk assessment, a series of risk reduction minisessions based on each couple's risk profile, interactive exercises, individual testing sessions, and couple-based posttest counseling; In woman-only relationship-focused HIV-CT group, the structure and content were matched as closely as possible with that of CB-HIV-CT (2) Standard-of-care: Administered to individuals in 2 sessions, including pretest counseling and voluntary HIV and hepatitis B and C testing and posttest results and risk reduction reinforcement	HIV educators or individuals with prior experience as outreach workers or HIV counselors	Central and East Harlem and South Bronx, New York, New York, USA	Weekly 2-h sessions for 6 weeks	As an intervention component, a short dyadic risk assessment was used to tailor each couple's risk profile and further customize to accommodate couples' stage of behavior change	Principal Investigator (PI): Randomly selected and monitored 10% of the interventions in each treatment condition to assess fidelity. Fidelity assessment included a checklist and monitor notes Supervisors: Discussed adherence to protocols and intervention fidelity with interventionists monthly	3-month follow-up retention rate: 83% (274/330) for women and 69% (228/330) for men
Remien et al. (2005) [35]	SMART Couples Study	Assess the efficacy of a couple-based intervention to improve medication-taking behavior in a clinic population Guiding theory: Social Action Theory	(1) Couple-based adherence intervention: Education about treatment and adherence, identifying adherence barriers, developing communication and problem-solving strategies, optimizing partner support, and building confidence for optimal adherence (2) Usual care: Standard clinical care provides attention to adherence-related issues from a multidisciplinary treatment team	A nurse practitioner	2 HIV/AIDS outpatient clinics in New York, New York, USA	4 sessions, 45–60 min each over 5 weeks	N/A	Interviewers: Received training and certificate to perform data collection Facilitators: Received training and satisfactorily completed pilot sessions before meeting with trial participants Supervisors and PI: Systematic reviews of interview audiotapes and weekly meetings with interviewers and facilitators	Week 32 study appointment assessment retention rate: Intervention: 83% (88/106), Control: 86% (94/109)
Sharma et al. (2020) [36]	The Unite for a Better Life (UBL) program	Reduce physical and sexual IPV and HIV risk behaviors as well as promote healthier, more equitable relationships Guiding theory not specified	UBL: A coffee ceremony in which 2 participants (Women/men/couples) prepared and served the coffee, and facilitators assisted discussion and interactive activities focused on gender norms, sexuality, communication and conflict resolution, HIV/AIDS, and IPV	Female and male facilitators	Rural Ethiopia in the context of the coffee ceremony	Twice-weekly 14 participatory and skills-building sessions (total 38 h)	N/A	Intervention coordinator: Observed sessions and provided ongoing feedback to facilitators to ensure intervention fidelity during implementation	Completion of intervention sessions (at least 85%): 72% in the couples' UBL arm, 85% in the women's UBL arm, and 62% in the men's UBL arm
Speizer et al. (2018) [37]	Couples' Health CoOp	Examine varying strategies to engage women and men around HIV prevention and im- proved couple interactions Guiding theory: Couple's Interdependence Theory	(1) WHC (male partners received HIV testing and counseling): Education on risk reduction strategies, extensively trained peer interventionists, skills training for risk reduction, role-playing on negotiating safe sex and communication skills, development of action plans to meet personalized goals, HIV testing, and referrals for other health services as necessary (2) CHC: Couple-level role-playing of risk reduction and communication skills and development of a joint action plan	N/A	A large Black African community out-side of the city center of Cape Town, South Africa	Two-session four-module program	Used the ADAPT framework to modify an existing, efficacious women's HIV prevention intervention to include components of an evidence-based couple's intervention from Project Connect and components from the Men as Partners program	N/A	Overall 6-month retention rate: 89.3% (267/299)
Sullivan et al. (2014) [38]	CHCT for US MSM	To evaluate the acceptability and safety of CHCT Guiding theory not specified	(1) CHCT: A couples counseling and testing service adapted for use with male couples (pretest discussion, HIV testing of both partners, skills-building around sexual agreements, return of HIV test results to both partners together, and posttest discussion) (2) iVCT: Standard prevention counseling and HIV testing Both arms complete a short questionnaire regarding satisfaction with the session at the end of the visit	A counselor trained in couple counseling strategies	A community-based organization (AID Atlanta), Atlanta, Georgia, USA	One-off CHCT/IVCT for 30–60 min	Adapted a CHCT service specifically for US MSM from the recommendation of the US President's Emergency Plan for AIDS Relief and CHCT guidelines of the World Health Organization	N/A	3-month follow-up retention rate: 79% (27/38) in CHCT and 92% (35/44) in iVCT

Item 3 What (Materials) was not included because it was not applicable for the intervention components

Item 6 How was not specified because all interventions were delivered in a face-to-face mode

Item 10 Modifications was not included as none of the studies reported related information

Different from Jiwatram-Negrón and El-Bassel’s categorization of couple-based HIV prevention and intervention biobehavioral studies [16], we identified two main HIV prevention intervention components: couple-based skills-building and couple-based HIV counseling and testing (CHCT). The couple-based skill-building component incorporated relationship-enhancing, risk-reduction, joint decision-making, and collaborative problem-solving skills, which also integrated with ART adherence education in one RCT study [35]. The CHCT component provided a single-session 30–60 min counseling and testing service based on couples’ gender roles, sexual orientation, sexual or drug use history, and risk assessment, or ethnicity. For instance, the CHCT component for male couples discussed HIV risks and how they wish to approach HIV prevention and skills-building around sexual agreements in the future [38]. From the individual-based control arms, we also differentiated two components: individual-based health education that promotes participants’ overall health or provides HIV/STD information, and individual-based usual care that covers standard clinical care providing ART medication adherence or standard HIV counseling and testing.

The intervention ranged from one to 14 sessions, apart from three interventions that offered a one-off CHCT session [28, 29, 38]. Six interventions were conducted with one session per week [30–34, 36]. Six studies tailored interventions to the couple’s dyadic risk features, findings from previous couple-based interventions, and characteristics of the key populations [29, 31, 32, 34, 37, 38]. However, modifications of intervention content or implementation procedure were not reported in any of the 11 studies. In addition, seven studies adopted strategies (e.g., fidelity check by on-site supervisor) to maintain or improve fidelity [30–36].

## Discussion

This meta-analysis is the first to synthesize existing RCTs, and it finds that couple-based interventions are more efficacious in promoting condom use and HIV testing (i.e., biobehavioral prevention) than individual-level interventions. As moderators for intervention effect, participants with higher education levels, couple-based interventions utilizing HIV testing and counseling strategies, and interventions conducted in low-income countries showed more significant improvement in condom use. This suggests that, despite the potential to reduce sexual risks and enhance HIV testing, many couple-based HIV prevention interventions conducted worldwide remain inadequate. Therefore, the following discussion also highlights significant methodological constraints of RCTs that are critical for facilitating advancements in couple-based HIV prevention interventions.

### Relative Effect of Couple-Based Interventions on HIV-Preventive Behaviors

This systematic review suggests that couple-based interventions have a stronger effect than individual-level interventions to boost condom use and encourage HIV testing among couples in RCTs. One possibility is that behavioral cooperation may help couples establish common goals in initiating healthy behaviors and developing a partnership based on joint decision-making and collective action toward reducing HIV transmission risks [13]. When both partners participate in couple-based interventions, they decrease discrepant perceptions of HIV transmission risks and increase their health literacy regarding HIV [39].

Another possible consideration is that enhanced communicative skills may enable couples to discuss complex or private sexual issues more openly. However, such inter-couple discussions are hard to implement without proper guidance [37, 40]. Couple-based intervention offers a promising platform to promote “open” discussions with a trained counselor or psychologist to help discuss sexual issues in a safe and constructive environment [41].

Communication-based intervention techniques may help couples to acknowledge sexual consent and shift their normative perceptions of their sexual relationships [42], thereby enhancing their dyadic resilience and relationship health as a whole [4]. In particular, interventions emphasizing a healthy sexuality, a healthy couple relationship, power-balancing, negotiating power, and joint decision-making may provide opportunities for cultivating open discussions. One example is that CHCT service offers a room for couples to learn about HIV risk for individuals and couples, to find out couples’ HIV status, and form a collaborative HIV prevention plan with help from a counselor who can facilitate couple discussion to reduce fears and possible negative consequences before and after disclosure [43, 44].

### Moderators of Intervention Effect in Promoting Condom Use

We identified three moderators of the intervention effect in promoting condom use: (1) intervention design incorporating HIV counseling and testing, (2) low- and middle-income countries, and (3) high school or above education level. First, HIV counseling and testing showed a stronger intervention effect than skills-building in promoting condom use. Although we did not conduct subgroup analyses on HIV testing due to limited comparisons, one 2020 RCT study found that the skills-building component of a couple-based intervention resulted in comparable effects on both condom use and HIV testing [36]. It is worth noting that interventions containing a skills-building strategy showed smaller, but significant, effects on condom use. Therefore, an additional question merits further examination: Will a combined-component couple-based intervention (i.e., CHCT plus skills-building) outperform a single-component couple-based intervention (i.e., CHCT or skills-building alone) in promoting more significant and longer-lasting effects on HIV-preventive behaviors? Some individual-level multi-component interventions may shed light on the answer to this question [45, 46]. For example, an RCT study demonstrated that MSM exposed to combined behavioral (e.g., interpersonal communication) and biomedical (e.g., HIV testing and counseling) components were more likely to use condoms during anal sex compared to those in the single-component group [45].

Second, adapting intervention material to participants’ education levels may increase the effectiveness of HIV transmission prevention interventions. As previously mentioned, participants with a high school education or above were more likely to use condoms than those with a lower education level. Intervention research that incorporates a skills-building component and typically takes several hours of training [31, 32, 47] may be too pedantically saturated to benefit participants with lower education levels. Therefore, we recommend a simplified version of the implementation protocols for participants with lower education levels that highlight the practical features of HIV transmission prevention and use layman’s language in combination with suggestions to practice behavioral and habitual changes at home.

Third, couple-based HIV prevention interventions may be more beneficial to reducing sexual risks when implemented in areas where resources are more constrained, such as in LMICs. Evidence from prior research supports our findings that CHCT has been recognized as one of the most cost-effective intervention strategies in resource-constrained settings and is particularly strong in HIV-serodiscordant couples [43]. This may be explained by the mobilizing of shared resources within a couple that were previously rarely considered, including the awareness of the importance of sexual negotiation, and supporting once-scarce environmental resources such as the space for an open and private conversation with a trained counselor for CHCT and/or access to PrEP [12, 13]. Although the evidence for cost-effectiveness analyses of skill-building intervention components remains to be clarified, future public health intervention strategies need to be weighted more toward resource-constrained areas with the objective to more effectively reduce HIV risk among couples.

### Methodological and Theoretical Barriers and Recommendations

Several methodological and theoretical barriers must be highlighted because they risk impacting the advancement of couple-based HIV prevention interventions. First, although study quality did not appear to moderate the intervention effect in RCTs, seven of the 11 studies (63.6%) failed to conceal allocation or blind the assessor. Consequently, a low-quality or inadequately designed study may still distort the verifiability of the study’s effects because of these two issues. We suggest that the allocation of quality assessment measures be executed by off-site third parties or by using sequentially numbered, sealed, opaque envelopes to establish better methodological and reporting quality.

Moreover, because eight of the 11 studies (72.7%) were published before 2014, only one study reported its intervention following the TIDieR checklist for better reporting and replication [36]. By utilizing TIDieR, we found that six out of 11 studies reported tailoring the intervention content to the study participants, mainly in risk-assessment and risk-reduction plans that seldom consider embedded sociocultural contexts.

Regrettably, most (six out of seven) of the reviewed couple-based HIV preventive intervention RCTs reported using theoretical frameworks were guided by individual-based theories focusing on behavioral changes at the individual level, such as social cognitive theory and combined ecological theory, rather than dyadic-level theories [31, 34]. As a result, many aspects involving dyads, interactions, and relationships were frequently overlooked in the theoretical models applied in the interventions [33, 35]. These irregularities in quality assessment signify that two improvements are needed for better reporting and replication across intervention studies, namely sociocultural sensitivity and theoretical context of relationship dynamics.

We first recommend that interventions adapt a more socioculturally sensitive approach by considering both partners’ cumulative education levels in coordination with other demographic characteristics of the local target populations, such as income level and sexual orientation. Although heterosexual couples are disproportionately represented in these 11 interventions, the target participants were often subgroups of populations impacted by low income, poor housing (including homelessness), or domestic violence [32, 33]. Moreover, only a few past and ongoing studies focus on partner concurrency [18] and male couples [38, 48], and even fewer on drug users and their sexual partners [32, 33] or transgender women and their sexual partners [49, 50]. The scarcity of couple-based RCTs in key populations has become a pressing research concern given the central role that HIV prevention plays in these key populations’ partnerships. Although researchers need to design intervention protocols with clear linkages to outcome variables, it is also critical to incorporate participants’ backgrounds and interpersonal relationships within intervention strategies and customize protocols to meet couple-based participants’ specific needs.

We also suggest that interventions may benefit from more thoroughly considering relationship dynamics in a broader theoretical context. One way to do this is to identify appropriate outcome measures based on the couple’s interdependence within dyadic coping perspectives [4]. To date, intervention studies have mainly used outcome measures and statistical methods to indicate individual-level behavioral change. Although researchers have made enormous efforts to design and implement core behavioral intervention components (e.g., skills-building for couples), none of the studies included in this review evaluated the dyadic outcomes, such as relationship quality [51] and sexual agreement [52] between partners. However, it is reassuring to note that in a recent dyadic intervention for improvement of HIV care engagement among HIV-serodiscordant male couples, Stephenson and colleagues (2017) [53] adopted a framework grounded in Couple’s Interdependence Theory [19] and selected dyadic measures of behavioral change within the couple.

Another method is to explore theories that integrate relationship dynamics and HIV prevention. There is a large gap between theories construing the relationship dynamics that may influence the transformation of motivation and health behavior, especially among same-sex couples in an HIV-serodiscordant relationship. A recent qualitative study revealed how “viral load agreements” facilitate the practice of different strategies in undetectable viral load for prevention among gay male couples [8], shedding new light on the development of theoretical constructs. Future RCTs may integrate renewed dyadic constructs into their interventions [54] following dyadic theoretical models such as the systemic transactional model [55] and the dyadic health model [56]. Researchers may also consider the relational dynamic characteristics of other key populations when tailoring interventions for the specific groups, such as interpersonal dynamics-based detoxification of injecting drug users [57], gender roles of transgender women [49], and intimate partner violence experienced by female sex workers [58].

### Limitations

There are some limitations to this study. First, a relatively small number of RCTs are available, limiting the statistical power for subgroup meta-analysis and meta-regression. Second, because all 11 studies were conducted in the USA and Africa, the conclusions drawn in this study may not be generalizable to, or adequately representative of, other world regions. Third, given that most studies have been exclusively targeted at heterosexual couples, there is an urgent need for more robust bodies of evidence on the effect of intervention studies on more diverse populations. In the future, with more original RCTs assessing intervention effects among key populations from more global perspectives, an updated meta-analysis would provide more evidence of the sustainable impact of couple-based interventions.

## Conclusions

In this systematic review and meta-analysis, couple-based interventions are more efficacious than individual-level interventions in biobehavioral HIV prevention. The intervention effect of couple-based HIV prevention RCTs will be improved by considering sociocultural sensitivities and theoretical contexts in relationship dynamics. Finally, couple-based HIV prevention RCTs are still in their infancy, and studies among key populations (i.e., MSM, injecting drug users, sex workers, and transgender women) warrant further investigation.

## Supplementary Information

Below is the link to the electronic supplementary material.

Supplementary file1 (DOCX 52 KB)

## Data Availability

The study was pre-registered at the PROSPERO database (CRD42020222819, https://www.crd.york.ac.uk/PROSPERO/). Materials used to conduct the study are publically available at Open Science Framework (Identifier: https://doi.org/10.17605/OSF.IO/7NBJK, https://osf.io/7nbjk/?view_only=eb5a91ea50b74c90b6999237c8795f85).
