# Supplementary material for: Do Couple-Based Interventions Show Larger Effects in Promoting HIV Preventive Behaviors than Individualized Interventions in Couples? A Systematic Review and Meta-analysis of 11 Randomized Controlled Trials
Source: AIDS Behav. 2022 Jul 15;27(1):314–34. doi: 10.1007/s10461-022-03768-5 (PMC10191920; doi:10.1007/s10461-022-03768-5)
Supplement: Supplementary file 1 — Supplementary file1 (DOCX 52 KB) [file 10461_2022_3768_MOESM1_ESM.docx]

# **Table A Preferred Reporting Items for Systematic Reviews and Meta-Analysis (PRISMA)**

| **Section/topic** | **#** | **Checklist item** | **Reported on page #** |
| --- | --- | --- | --- |
| **TITLE** | | |  |
| Title | 1 | Identify the report as a systematic review, meta-analysis, or both. | 1 |
| **ABSTRACT** | | |  |
| Structured summary | 2 | Provide a structured summary including, as applicable: background; objectives; data sources; study eligibility criteria, participants, and interventions; study appraisal and synthesis methods; results; limitations; conclusions and implications of key findings; systematic review registration number. | 1 |
| **INTRODUCTION** | | |  |
| Rationale | 3 | Describe the rationale for the review in the context of what is already known. | 2 |
| Objectives | 4 | Provide an explicit statement of questions being addressed with reference to participants, interventions, comparisons, outcomes, and study design (PICOS). | 3 |
| **METHODS** | | |  |
| Protocol and registration | 5 | Indicate if a review protocol exists, if and where it can be accessed (e.g., Web address), and, if available, provide registration information including registration number. | 3 |
| Eligibility criteria | 6 | Specify study characteristics (e.g., PICOS, length of follow-up) and report characteristics (e.g., years considered, language, publication status) used as criteria for eligibility, giving rationale. | 4-5 |
| Information sources | 7 | Describe all information sources (e.g., databases with dates of coverage, contact with study authors to identify additional studies) in the search and date last searched. | 4 |
| Search | 8 | Present full electronic search strategy for at least one database, including any limits used, such that it could be repeated. | 4 |
| Study selection | 9 | State the process for selecting studies (i.e., screening, eligibility, included in systematic review, and, if applicable, included in the meta-analysis). | 4-5 |
| Data collection process | 10 | Describe method of data extraction from reports (e.g., piloted forms, independently, in duplicate) and any processes for obtaining and confirming data from investigators. | 5 |
| Data items | 11 | List and define all variables for which data were sought (e.g., PICOS, funding sources) and any assumptions and simplifications made. | 5 |
| Risk of bias in individual studies | 12 | Describe methods used for assessing risk of bias of individual studies (including specification of whether this was done at the study or outcome level), and how this information is to be used in any data synthesis. | 6-7 |
| Summary measures | 13 | State the principal summary measures (e.g., risk ratio, difference in means). | 6-7 |
| Synthesis of results | 14 | Describe the methods of handling data and combining results of studies, if done, including measures of consistency (e.g., I^2^) for each meta-analysis. | 7-8 |

# **Table B Full Search Strategy**

| Electronic Databases | Search term | Search date |
| --- | --- | --- |
| Primary searches | | |
| PubMed | Participant keywords (Couple OR dyad OR partner OR married) in title and abstract AND in clinical trials and randomized controlled trials  Intervention-related terms (intervention OR prevention) in title and abstract AND in clinical trials and randomized controlled trials  Disease-related terms (HIV OR AIDS) in title and abstract AND in clinical trials and randomized controlled trials | 2020.11.30 |
| PsycINFO | Participant keywords (Couple OR dyad OR partner OR married) in title and abstract  Intervention-related terms (intervention OR prevention OR randomized control trials OR randomized clinical trial) in title and abstract  Disease-related terms (HIV/AIDS) in title and abstract | 2020.12.3 |
| CINAHL | Participant keywords (Couple OR dyad OR partner OR married) in abstract  Intervention-related terms (intervention OR prevention) in abstract  Disease-related terms (HIV/AIDS) in abstract | 2020.12.5 |
| Web of science | Participant keywords (Couple OR dyad OR partner) in title and abstract  Intervention-related terms (intervention OR prevention OR randomized control trials OR randomized clinical trial) in title and abstract  Disease-related terms (HIV OR AIDS) in title and abstract | 2020.12.19 |
| Supplementary searches | | |
| Clinicaltrials.gov | Participant keywords (Couple OR dyad) in clinical trials completed with results  Intervention-related terms (intervention OR prevention) in clinical trials completed with results  Disease-related terms (HIV OR AIDS) in clinical trials completed with results | 2020.12.8 |
| Previous published systematic review and meta-analysis | Burton J, Darbes LA, Operario D. Couples-focused behavioral interventions for prevention of HIV: Systematic review of the state of evidence. *AIDS Behav*. 2010;14(1):1-10.  LaCroix JM, Pellowski JA, Lennon CA, Johnson BT. Behavioural interventions to reduce sexual risk for HIV in heterosexual couples: A meta-analysis. *Sex Transm Infect*. 2013;89(8):620-627.  Jiwatram-Negrón T, El-Bassel N. Systematic review of couple-based HIV intervention and prevention studies: Advantages, gaps, and future directions. *AIDS Behav*. 2014;18(10):1864-1887.  Crepaz N, Tungol-Ashmon MV, Vosburgh HW, Baack BN, Mullins MM. Are couple-based interventions more effective than interventions delivered to individuals in promoting HIV protective behaviors? A meta-analysis. *AIDS Care*. 2015;27(11):1361-1366. | 2020.11.30  2020.12.5  2020.12.19 |

# **Table C Reasons for Excluding in Full-text Assessment**

| No. | Full text being excluded | Reasons |
| --- | --- | --- |
| 1 | Allen S, Tice J, Van de Perre P, et al. Effect of serotesting with counselling on condom use and seroconversion among HIV discordant couples in Africa. *BMJ*. 1992;304(6842):1605-1609. | Not an RCT design |
| 2 | Baeten JM, Donnell D, Ndase P, et al. Antiretroviral prophylaxis for HIV prevention in heterosexual men and women. *N Engl J Med*. 2012;367(5):399-410. | No couple vs. individual comparison |
| 3 | Becker S, Taulo FO, Hindin MJ, Chipeta EK, Loll D, Tsui A. Pilot study of home-based delivery of HIV testing and counseling and contraceptive services to couples in Malawi. *BMC Public Health*. 2014;14:1309. | Not an RCT design |
| 4 | Belus JM, Baucom DH, Carney T, Carrino EA, Wechsberg WM. A South African couple-based HIV prevention program: Preliminary evidence of the long-term effects. *J Assoc Nurses AIDS Care*. 2019;30(6):648-657. | Not an RCT design |
| 5 | Bent-Goodley T. In Circle: A healthy relationship, domestic violence, and HIV intervention for African American couples. *J Hum Behav Soc Environ*. 2017;27(1-2):132-140. | Not an RCT design |
| 6 | Bouris A, Jaffe K, Eavou R, et al. Project nGage: Results of a randomized controlled trial of a dyadic network support intervention to retain young black men who have sex with men in HIV care. *AIDS Behav*. 2017;21(12):3618-3629. | No intervention or control group is couple-based or couple-focused |
| 7 | Byamugisha R, Åstrøm AN, Ndeezi G, Karamagi CAS, Tylleskär T, Tumwine JK. Male partner antenatal attendance and HIV testing in eastern Uganda: A randomized facility-based intervention trial. *J Int AIDS Soc*. 2011;14(1):43. | No intervention or control group is couple-based or couple-focused |
| 8 | Celum C, Wald A, Lingappa JR, et al. Acyclovir and transmission of HIV-1 from persons infected with HIV-1 and HSV-2. *N Engl J Med*. 2010;362(5):427-439. | No couple vs. individual comparison |
| 9 | Efficacy of voluntary HIV-1 counselling and testing in individuals and couples in Kenya, Tanzania, and Trinidad: A randomised trial. The Voluntary HIV-1 Counseling and Testing Efficacy Study Group. *Lancet*. 2000;356(9224):103-112. | No couple vs. individual comparison |
| 10 | Cohen MS, Chen YQ, McCauley M, et al. Prevention of HIV-1 infection with early antiretroviral therapy. *N Engl J Med*. 2011;365(6):493-505. | No couple vs. individual comparison |
| 11 | Choko AT, Corbett EL, Stallard N, et al. HIV self-testing alone or with additional interventions, including financial incentives, and linkage to care or prevention among male partners of antenatal care clinic attendees in Malawi: An adaptive multi-arm, multi-stage cluster randomised trial. *PLoS Med*. 2019;16(1):e1002719. | No intervention or control group is couple-based or couple-focused |
| 12 | Darbes LA, McGrath NM, Hosegood V, et al. Results of a couples-based randomized controlled trial aimed to increase testing for HIV. *J Acquir Immune Defic Syndr*. 2019;80(4):404-413. | No couple vs. individual comparison |
| 13 | Ditekemena J, Matendo R, Koole O, et al. Male partner voluntary counselling and testing associated with the antenatal services in Kinshasa, Democratic Republic of Congo: A randomized controlled trial. *Int J STD AIDS*. 2011;22(3):165-170. | No intervention or control group is couple-based or couple-focused |
| 14 | El-Bassel N, Witte SS, Gilbert L, et al. Long-term effects of an HIV/STI sexual risk reduction intervention for heterosexual couples. *AIDS Behav*. 2005;9(1):1-13. | The same study as El-Bassel et al., 2003 |
| 15 | El-Bassel N, Jemmott JB 3rd, Landis JR, et al. Intervention to influence behaviors linked to risk of chronic diseases: A multisite randomized controlled trial with African-American HIV-serodiscordant heterosexual couples. *Arch Intern Med*. 2011;171(8):728-736. | The same study as El-Bassel et al., 2010 |
| 16 | El-Bassel N, Gilbert L, Terlikbayeva A, et al. Effects of a couple-based intervention to reduce risks for HIV, HCV, and STIs among drug-involved heterosexual couples in Kazakhstan: A randomized controlled trial. *J Acquir Immune Defic Syndr*. 2014;67(2):196-203. | No couple vs. individual comparison |
| 17 | El-Bassel N, Gilbert L, Goddard-Eckrich D, et al. Effectiveness of a couple-based HIV and sexually transmitted infection prevention intervention for men in community supervision programs and their female sexual partners: A randomized clinical trial. *JAMA Netw Open*. 2019;2(3):e191139. | No couple vs. individual comparison |
| 18 | Gilbert L, El-Bassel N, Terlikbayeva A, et al. Couple-based HIV prevention for injecting drug users in Kazakhstan: A pilot intervention study. *J Prev Interv Community*. 2010;38(2):162-176. | No couple vs. individual comparison |
| 19 | Ezeanolue EE, Obiefune MC, Yang W, et al. What do you need to get male partners of pregnant women tested for HIV in resource limited settings? The baby shower cluster randomized trial. *AIDS Behav*. 2017;21(2):587-596. | No intervention or control group is couple-based or couple-focused |
| 20 | Fife BL, Scott LL, Fineberg NS, Zwickl BE. Promoting adaptive coping by persons with HIV disease: Evaluation of a patient/partner intervention model. *J Assoc Nurses AIDS Care*. 2008;19(1):75-84. | Study outcomes are not related to sexual-risk reduction |
| 21 | Gilbert L, Hunt T, Primbetova S, et al. Reducing opioid overdose in Kazakhstan: A randomized controlled trial of a couple-based integrated HIV/HCV and overdose prevention intervention "Renaissance". *Int J Drug Policy*. 2018;54:105-113. | The same study as El-Bassel et al., 2014 |
| 22 | Gordon-Garofalo VL, Rubin A. Evaluation of a psychoeducational group for seronegative partners and spouses of persons with HIV/AIDS. *Res Soc Work Pract*. 2004;14(1):14-26. | Study outcomes are not related to HIV/AIDS |
| 23 | Gross R, Zheng L, La Rosa A, et al. Partner-based adherence intervention for second-line antiretroviral therapy (ACTG A5234): A multinational randomised trial. *Lancet HIV*. 2015;2(1):e12-e19. | Study outcomes are not related to sexual-risk reduction |
| 24 | Harrison SE, Li X, Zhang J, Chi P, Zhao J, Zhao G. Improving school outcomes for children affected by parental HIV/AIDS: Evaluation of the ChildCARE Intervention at 6-, 12-, and 18-months. *Sch Psychol Int*. 2017;38(3):264-286. | Study participants are not in a sexual relationship |
| 25 | Harvey SM, Henderson JT, Thorburn S, et al. A randomized study of a pregnancy and disease prevention intervention for Hispanic couples. *Perspect Sex Reprod Health*. 2004;36(4):162-169. | No couple vs. individual comparison |
| 26 | Hatcher AM, Darbes L, Kwena Z, et al. Pathways for HIV prevention behaviors following a home-based couples intervention for pregnant women and male partners in Kenya. *AIDS Behav*. 2020;24(7):2091-2100. | Secondary analysis |
| 27 | Javalkar P, Platt L, Prakash R, et al. Effectiveness of a multilevel intervention to reduce violence and increase condom use in intimate partnerships among female sex workers: Cluster randomised controlled trial in Karnataka, India. *BMJ Glob Health*. 2019;4(6):e001546. | No intervention or control group is couple-based or couple-focused |
| 28 | Jones D, Kashy D, Chitalu N, et al. Risk reduction among HIV-seroconcordant and -discordant couples: The Zambia NOW2 intervention. *AIDS Patient Care STDS*. 2014;28(8):433-441. | No intervention or control group is couple-based or couple-focused |
| 29 | Kajubi P, Ruark A, Hearst N, Ruteikara S, Green EC. Assessment of an HIV-prevention intervention for couples in peri-urban Uganda: Pervasive challenges to relationship quality also challenge intervention effectiveness. *Afr J AIDS Res*. 2020;19(3):249-262. | Not an RCT design |
| 30 | Koniak-Griffin D, Lesser J, Henneman T, et al. HIV prevention for Latino adolescent mothers and their partners. *West J Nurs Res*. 2008;30(6):724-742. | Not an RCT design |
| 31 | Koniak-Griffin D, Lesser J, Takayanagi S, Cumberland WG. Couple-focused human immunodeficiency virus prevention for young Latino parents: Randomized clinical trial of efficacy and sustainability. *Arch Pediatr Adolesc Med*. 2011;165(4):306-312. | No couple vs. individual comparison |
| 32 | Krakowiak D, Kinuthia J, Osoti AO, et al. Home-based HIV testing among pregnant couples increases partner testing and identification of serodiscordant partnerships. *J Acquir Immune Defic Syndr*. 2016;72 Suppl 2(Suppl 2):S167-S173. | No couple vs. individual comparison |
| 33 | Lesser J, Koniak-Griffin D, Gonzalez-Figueroa E, Huang R, Cumberland WG. Childhood abuse history and risk behaviors among teen parents in a culturally rooted, couple-focused HIV prevention program. *J Assoc Nurses AIDS Care*. 2007;18(2):18-27. | Provide baseline data only |
| 34 | Lyon ME, Squires L, D’Angelo LJ, et al. FAmily-CEntered (FACE) advance care planning among African-American and non-African-American adults living with HIV in Washington, DC: A randomized controlled trial to increase documentation and health equity. *J Pain Symptom Manage*. 2019;57(3):607-616. | Study participants are not in a sexual relationship |
| 35 | Maman S, Mulawa MI, Balvanz P, et al. Results from a cluster-randomized trial to evaluate a microfinance and peer health leadership intervention to prevent HIV and intimate partner violence among social networks of Tanzanian men. *PLoS One*. 2020;15(3):e0230371. | No intervention or control group is couple-based or couple-focused |
| 36 | Mark J, Kinuthia J, Roxby AC, et al. Uptake of home-based syphilis and human immunodeficiency virus testing among male partners of pregnant women in Western Kenya. *Sex Transm Dis*. 2017;44(9):533-538. | No intervention or control group is couple-based or couple-focused |
| 37 | Mark J, Kinuthia J, Osoti AO, et al. Male partner linkage to clinic-based services for sexually transmitted infections and human immunodeficiency virus services following couple home-based education and testing. *Sex Transm Dis*. 2019;46(11):716-721. | Secondary analysis |
| 38 | Martinez O, Wu E, Levine EC, et al. Integration of social, cultural, and biomedical strategies into an existing couple-based behavioral HIV/STI prevention intervention: Voices of Latino male couples. *PLoS One*. 2016;11(3):e0152361. | Not an RCT design |
| 39 | Martinez O, Wu E, Frasca T, et al. Adaptation of a couple-based HIV/STI prevention intervention for Latino men who have sex with men in New York City. *Am J Mens Health*. 2017;11(2):181-195. | Not an RCT design |
| 40 | Marwa T, Karanja S, Osero J, Orago A. The effects of HIV self-testing kits in increasing uptake of male partner testing among pregnant women attending antenatal clinics in Kenya: A randomized controlled trial. *Pan Afr Med J*. 2019;33:213. | No intervention or control group is couple-based or couple-focused |
| 41 | Mashaphu S, Wyatt GE, Zhang M, Mthiyane T, Liu H, Gomo E. Effectiveness of an HIV-risk reduction intervention to reduce HIV transmission among serodiscordant couples in Durban, South Africa: A randomized controlled trial. *AIDS Care*. 2020;32(5):537-545. | No intervention or control group is couple-based or couple-focused |
| 42 | Matovu JK, Todd J, Wanyenze RK, Kairania R, Serwadda D, Wabwire-Mangen F. Evaluation of a demand-creation intervention for couples' HIV testing services among married or cohabiting individuals in Rakai, Uganda: A cluster-randomized intervention trial. *BMC Infect Dis*. 2016;16:379. | No intervention or control group is couple-based or couple-focused |
| 43 | McMahon JM, Pouget ER, Tortu S, Volpe EM, Torres L, Rodriguez W. Couple-based HIV counseling and testing: A risk reduction intervention for US drug-involved women and their primary male partners. *Prev Sci*. 2015;16(2):341-351. | The same study as McMahon et al., 2013 |
| 44 | McMahon JM, Chimenti R, Trabold N, Fedor T, Mittal M, Tortu S. Risk of intimate partner violence and relationship conflict following couple-based HIV prevention counseling: Results from the Harlem River Couples Project. *J Interpers Violence*. 2017;32(24):3709-3734. | The same study as McMahon et al., 2013 |
| 45 | Miller KS, Lin CY, Poulsen MN, et al. Enhancing HIV communication between parents and children: Efficacy of the Parents Matter! Program. *AIDS Educ Prev*. 2011;23(6):550-563. | Study participants are not in a sexual relationship |
| 46 | Mitchell JW, Lee JY, Wu Y, Sullivan PS, Stephenson R. Feasibility and acceptability of an electronic health HIV prevention toolkit intervention with concordant HIV-negative, same-sex male couples on sexual agreement outcomes: Pilot randomized controlled trial. *JMIR Form Res*. 2020;4(2):e16807. | Study outcomes are not related to sexual-risk reduction |
| 47 | Nkhoma K, Seymour J, Arthur A. An educational intervention to reduce pain and improve pain management for Malawian people living With HIV/AIDS and their family carers: A randomized controlled trial. *J Pain Symptom Manage*. 2015;50(1):80-90.e4. | Study participants are not in a sexual relationship |
| 48 | Operario D, Gamarel KE, Iwamoto M, et al. Couples-focused prevention program to reduce HIV risk among transgender women and their primary male partners: Feasibility and promise of the couples HIV intervention program. *AIDS Behav*. 2017;21(8):2452-2463. | No couple vs. individual comparison |
| 49 | Osoti AO, John-Stewart G, Kiarie J, et al. Home visits during pregnancy enhance male partner HIV counselling and testing in Kenya: A randomized clinical trial. *AIDS*. 2014;28(1):95-103. | Study outcomes are not related to or sexual-risk reduction |
| 50 | Pakenham KI, Dadds MR, Lennon HV. The efficacy of a psychosocial intervention for HIV/AIDS caregiving dyads and individual caregivers: A controlled treatment outcome study. *AIDS Care*. 2002;14(6):731-750. | Study participants are not in a sexual relationship |
| 51 | Peragallo Montano N, Cianelli R, Villegas N, Gonzalez-Guarda R, Williams WO, de Tantillo L. Evaluating a culturally tailored HIV risk reduction intervention among Hispanic women delivered in a real-world setting by community agency personnel. *Am J Health Promot*. 2019;33(4):566-575. | No intervention or control group is couple-based or couple-focused |
| 52 | Pomeroy EC, Green DL, Van Laningham L. Couples who care: The effectiveness of a psychoeducational group intervention for HIV serodiscordant couples. *Res Soc Work Pract*. 2002;12(2):238-252. | Not an RCT design |
| 53 | Rosenberg NE, Mtande TK, Saidi F, et al. Recruiting male partners for couple HIV testing and counselling in Malawi's option B+ programme: An unblinded randomised controlled trial. *Lancet HIV*. 2015;2(11):e483-e491. | No intervention or control group is couple-based or couple-focused |
| 54 | Schiff M, Witte SS, El-Bassel N. Client satisfaction and perceived helping components of an HIV/AIDS preventive intervention for urban couples. *Res Soc Work Pract*. 2003;13(4):468-492. | Study outcomes are not related to sexual-risk reduction |
| 55 | Settergren SK, Mujaya S, Rida W, et al. Cluster randomized trial of comprehensive gender-based violence programming delivered through the HIV/AIDS program platform in Mbeya Region, Tanzania: Tathmini GBV study. *PLoS One*. 2018;13(12):e0206074. | No intervention or control group is couple-based or couple-focused |
| 56 | Sibanda EL, Tumushime M, Mufuka J, et al. Effect of non-monetary incentives on uptake of couples’ counselling and testing among clients attending mobile HIV services in rural Zimbabwe: A cluster-randomised trial. *Lancet Glob Health*. 2017;5(9):e907-e915. | No intervention or control group is couple-based or couple-focused |
| 57 | Sifunda S, Peltzer K, Rodriguez VJ, et al. Impact of male partner involvement on mother-to-child transmission of HIV and HIV-free survival among HIV-exposed infants in rural South Africa: Results from a two phase randomised controlled trial. *PLoS One*. 2019;14(6):e0217467. | No intervention or control group is couple-based or couple-focused |
| 58 | Starks TJ, Dellucci TV, Gupta S, et al. A Pilot Randomized trial of intervention Components Addressing drug use in couples HIV testing and counseling (CHTC) with male couples. *AIDS Behav*. 2019;23(9):2407-2420. | No couple vs. individual comparison |
| 59 | Stephenson R, Mendenhall E, Muzizi L, et al. The influence of motivational messages on future planning behaviors among HIV concordant positive and discordant couples in Lusaka, Zambia. *AIDS Care*. 2008;20(2):150-160. | Study outcomes are not related to sexual-risk reduction |
| 60 | Tan JY, Campbell CK, Conroy AA, Tabrisky AP, Kegeles S, Dworkin SL. Couple-level dynamics and multilevel challenges among Black men who have sex with men: A framework of dyadic HIV Care. *AIDS Patient Care STDS*. 2018;32(11):459-467. | Not an RCT design |
| 61 | Turan JM, Darbes LA, Musoke PL, et al. Development and piloting of a home-based couples intervention during pregnancy and postpartum in Southwestern Kenya. *AIDS Patient Care STDS*. 2018;32(3):92-103. | No couple vs. individual comparison |
| 62 | Wawer MJ, Makumbi F, Kigozi G, et al. Circumcision in HIV-infected men and its effect on HIV transmission to female partners in Rakai, Uganda: A randomised controlled trial. *Lancet*. 2009;374(9685):229-237. | No intervention or control group is couple-based or couple-focused |
| 63 | Wechsberg WM, El-Bassel N, Carney T, Browne FA, Myers B, Zule WA. Adapting an evidence-based HIV behavioral intervention for South African couples. *Subst Abuse Treat Prev Policy*. 2015;10:6. | Not an RCT design |
| 64 | Weir BW, O'Brien K, Bard RS, et al. Reducing HIV and partner violence risk among women with criminal justice system involvement: A randomized controlled trial of two motivational interviewing-based interventions. *AIDS Behav*. 2009;13(3):509-522. | No intervention or control group is couple-based or couple-focused |
| 65 | Witte SS, Wu E, El-Bassel N, et al. Implementation of a couple-based HIV prevention program: A cluster randomized trial comparing manual versus Web-based approaches. *Implement Sci*. 2014;9:116. | Study outcomes are not related to sexual-risk reduction |
| 66 | Witte SS, El-Bassel N, Gilbert L, Wu E, Chang M, Hill J. Promoting female condom use to heterosexual couples: Findings from a randomized clinical trial. *Perspect Sex Reprod Health*. 2006;38(3):148-154. | The same study as El-Bassel et al., 2003 |
| 67 | Wu E, El-Bassel N, Donald McVinney L, Fontaine YM, Hess L. Adaptation of a couple-based HIV intervention for methamphetamine-involved African American men who have sex with men. *Open AIDS J*. 2010;4:123-131. | Not an RCT design |
| 68 | Wu E, El-Bassel N, McVinney LD, et al. Feasibility and promise of a couple-based HIV/STI preventive intervention for methamphetamine-using, black men who have sex with men. *AIDS Behav*. 2011;15(8):1745-1754. | Not an RCT design |
| 69 | Zhang J, Cederbaum JA, Jemmott JB 3rd, Jemmott LS. Theory-based behavioral intervention increases mother-son communication about sexual risk reduction among inner-city African-Americans. *J Adolesc Health*. 2018;63(4):497-502. | Study participants are not in a sexual relationship |
| 70 | Clinicaltrials.gov identifier: NCT02085356  Implementing a comprehensive prevention of mother-to-child transmission of HIV program for South African couples | Study participants are not in a sexual relationship |
| 71 | Clinicaltrials.gov identifier: NCT00557245  Pre-exposure prophylaxis to prevent HIV-1 acquisition within HIV-1 discordant couples (Partners PrEP) | Only HIV-negative partners are randomized |
| 72 | Clinicaltrials.gov identifier: NCT00074581  Preventing sexual transmission of HIV with anti-HIV drugs | No intervention or control group is couple-based or couple-focused |
| 73 | Clinicaltrials.gov identifier: NCT02139176  Assessing strategies for increasing male involvement in Malawi's antenatal program | No intervention or control group is couple-based or couple-focused |
| 74 | Clinicaltrials.gov identifier: NCT02043015  Comprehensive HIV prevention package for MSM in Southern Africa: Pilot study (Sibanye Health Project) | No intervention or control group is couple-based or couple-focused |
| 75 | Clinicaltrials.gov identifier: NCT00976404  Therapeutic intensification plus immunomodulation to decrease the HIV-1 viral reservoir | No intervention or control group is couple-based or couple-focused |
| 76 | Clinicaltrials.gov identifier: NCT02429869  Impact of everolimus on HIV persistence post kidney or liver transplant | No intervention or control group is couple-based or couple-focused |
| 77 | Clinicaltrials.gov identifier: NCT03076359  Traditional healers as adherence partners for persons living with HIV in rural Mozambique | Study participants are not in a sexual relationship |
| 78 | Clinicaltrials.gov identifier: NCT02334215  A randomized trial of interim methadone and patient navigation initiated in jail | No intervention or control group is couple-based or couple-focused |
| 79 | Clinicaltrials.gov identifier: NCT00218335  A network & dyad HIV prevention intervention for IDU's - 1 | No intervention or control group is couple-based or couple-focused |
| 80 | Clinicaltrials.gov identifier: NCT02021565  Evaluating the CG ASSIST program for caregiving dyads | No intervention or control group is couple-based or couple-focused |
| 81 | Clinicaltrials.gov identifier: NCT03463941  Peer support dyads in churches | Study participants are not in a sexual relationship |
| 82 | Clinicaltrials.gov identifier: NCT01822366  Randomized controlled trial of trauma-focused CBT in Tanzania and Kenya | No intervention or control group is couple-based or couple-focused |

**Table D Study Quality and Risk-of-bias for Included Studies**

| Study name | Specified eligibility criteria | Randomization | Concealed allocation | Similar baseline | Blinding of assessors | Retention> 85% | Intention-to-treat (ITT) | Between-group statistical comparisons | Point measures and variability | Total Score |
| --- | --- | --- | --- | --- | --- | --- | --- | --- | --- | --- |
| Becker et al., 2010 | 1 | 1 | 0 | 1 | 0 | 1 | 1 | 1 | 0 | 6 |
| Coates et al., 2000 | 1 | 1 | 1 | 1 | 0 | 1 | 1 | 1 | 1 | 8 |
| El-Bassel et al., 2003 | 1 | 1 | 1 | 1 | 0 | 1 | 1 | 1 | 1 | 8 |
| El-Bassel et al., 2010 | 1 | 1 | 1 | 1 | 1 | 1 | 1 | 1 | 1 | 9 |
| El-Bassel et al., 2011 | 1 | 1 | 0 | 1 | 0 | 1 | 1 | 1 | 1 | 7 |
| Jones et al., 2013 | 1 | 1 | 0 | 1 | 0 | 1 | 0 | 1 | 1 | 6 |
| McMahon et al.,2013 | 1 | 1 | 0 | 1 | 0 | 1 | 1 | 1 | 1 | 7 |
| Remien et al., 2005 | 1 | 1 | 0 | 1 | 1 | 1 | 1 | 1 | 1 | 8 |
| Sharma et al., 2020 | 1 | 1 | 1 | 1 | 1 | 1 | 1 | 1 | 1 | 9 |
| Speizer et al.,2018 | 1 | 1 | 0 | 1 | 0 | 1 | 0 | 1 | 1 | 6 |
| Sullivan et al., 2014 | 1 | 1 | 1 | 1 | 1 | 0 | 0 | 1 | 1 | 7 |
